# Anticipated and imagined futures: prospective cognition and depressed mood following brain injury

**DOI:** 10.1111/bjc.12202

**Published:** 2018-08-21

**Authors:** Fionnuala C. Murphy, Polly V. Peers, Simon E. Blackwell, Emily A. Holmes, Tom Manly

**Affiliations:** ^1^ Medical Research Council Cognition and Brain Sciences Unit Cambridge UK; ^2^ Department of Psychology Mental Health Research and Treatment Center Ruhr‐Universität Bochum Bochum Germany; ^3^ Department for Clinical Neuroscience Karolinska Institutet Stockholm Sweden

**Keywords:** depression, cognition, cognitive bias, prospective cognition, executive function, acquired brain injury

## Abstract

**Objectives:**

Depression, which is common following acquired brain injury (ABI), has been shown to predict cognitive impairment, rehabilitation outcome, and quality of life. Whilst many studies have examined links between depression and cognitive–affective processing in the non‐ABI population, their applicability to this important clinical group, where cognitive difficulties can be marked, remains unknown. Here, we investigated biases in prospective cognition, which is known to be disrupted in (non‐ABI) depression yet important for well‐being.

**Design:**

Cross‐sectional design with three groups (depressed ABI, non‐depressed ABI, and non‐ABI control participants). Continuous data were additionally analysed in correlation analyses.

**Methods:**

Individuals with ABI varying in extent of self‐reported depression and matched non‐ABI control participants completed assessments of mood and prospective cognition (anticipating and imagining future events), alongside background tests of executive function and fluid intelligence.

**Results:**

Relative to non‐depressed ABI and control participants, depressed ABI individuals demonstrated a reduced positive bias in prospective cognition: whereas non‐depressed ABI and control participants generated more examples of likely or possible positive versus negative future events, there was no evidence for such a positive bias in depressed ABI participants. Non‐depressed ABI and control participants also reported more vivid mental imagery for positive versus negative future scenarios, whereas such a pattern was not evident in depressed ABI participants. This pattern emerged despite background impairments in fluid intelligence and executive function associated with ABI.

**Conclusions:**

These findings (1) elucidate depression‐linked cognitive–affective processes following ABI, where cognitive difficulties are common, and (2) highlight psychological processes associated with depression that are common to ABI and non‐ABI populations.

**Practitioner points:**

***Clinical implications***

- A relative negative bias in future‐directed cognition is associated with depressed mood in individuals with chronic ABI.
- Such processes may contribute to the onset and maintenance of depression following ABI.
- These findings suggest it may be important to consider a role for prospective cognition in psychological interventions for depression following ABI.

***Limitations of the study***

- The extent to which depressed mood following ABI is associated with biases in other cognitive domains remains unclear.
- Whether similar patterns would be observed in acute patients with more profound cognitive difficulties requires further investigation.
- Despite large effect sizes, our sample size is modest; these effects thus require replication in larger groups.

Depressed mood is common following acquired brain injury (ABI) – injuries caused by stroke, tumour, traumatic collision, or skull‐penetrating objects. For example, approximately one‐third of over 25,000 stroke survivors report experiencing significant depression (Hackett & Pickles, 2014), with a higher frequency in some studies (Gainotti, Azzoni, & Marra, 1999; Kauhanen *et al*., 1999). Following brain tumour, 30% of patients individuals meet criteria for major depression, a rate higher than that found following other tumours (Wellisch, Kaleita, Freeman, Coughesy, & Goldman, 2002). And meta‐analysis indicates clinically significant depression in 38% of individuals following traumatic brain injury (TBI; Osborn, Mathias, & Fairweather‐Schmidt, 2014).

Acquired brain injury can have huge costs for individuals, families, and society. Stroke, for example, the largest cause of long‐term disability in many developed nations, can cause persistent physical, perceptual, and cognitive problems (Lloyd‐Jones *et al*., 2010; Saka, McGuire, & Wolfe, 2009). Added to this, depression can have further consequences for recovery and outcome, with post‐stroke depression linked to elevated risk of subsequent stroke and mortality (Dong, Zhang, Tong, & Qin, 2012; Pan, Sun, Okereke, Rexrode, & Hu, 2011), increased cognitive impairment (Bour, Rasquin, Limburg, & Verhey, 2011; Morris, Raphael, & Robinson, 1992), and poor outcome and quality of life (QoL; Carod‐Artal, Egido, Gonzalez, & de Seijas, 2000; Morris *et al*., 1992; Schmid *et al*., 2011). The picture for TBI is similar, with associations between depression and reduced QoL at 1 year despite adjustment for variables that predict depression (Bombardier *et al*., 2010). Up to 10 years post‐ABI, survivors with depression are up to 19 more times more likely to report hopelessness, poor concentration, and reduced enjoyment of life's activities (Seel *et al*., 2003). Effective interventions that target low mood post‐ABI are thus necessary (Wilson, 2008).

An important first step is to consider whether cognitive–affective processes implicated in depression in non‐ABI populations are similarly evident in ABI. In ‘healthy’ individuals who are depressed or at risk, biases in cognitive–affective processing have been identified that are now considered central to depression's onset and maintenance (Mathews & MacLeod, 2005) – in attention (Mccabe & Gotlib, 1993), memory (Harmer *et al*., 2009), interpretation (Wenzlaff & Bates, 1998), and executive control (Murphy, Dunn, Macpherson, Manly, & Jeyabalasingham, 2013; Murphy, Michael, & Sahakian, 2012; Murphy *et al*., 1999). For example, depressed individuals, relative to non‐depressed controls, have poorer memory for positive relative to negative information (Harmer *et al*., 2009). Findings like these have led to the development of novel, empirically derived cognitive training approaches that target these biases, with consequent improvements in mood (Blackwell & Holmes, 2010; Williams *et al*., 2015; Yiend *et al*., 2014). However, the extent to which depression‐linked biases in cognitive–affective processing are implicated in ABI, where wide‐ranging deficits in attention, memory, and executive functions are common, remains unknown.

Here, we specifically examine whether depressed mood following ABI is characterized by a relative negative bias in future cognition, and in particular, anticipating and imagining future events. In the non‐ABI population, where being able to anticipate and effectively simulate (‘pre‐experience’) events in the future is important for well‐being, the positive future bias typically observed in non‐depressed individuals is reduced or absent in those who are depressed or dysphoric (Holmes, Blackwell, Heyes, Renner, & Raes, 2016; MacLeod & Byrne, 1996; MacLeod, Tata, Kentish, & Jacobsen, 1997; Morina, Deeprose, Pusowski, Schmid, & Holmes, 2011).

The recruitment of common neural substrates during mental imagery (perceptual experience that occurs in the absence of sensory input, such as seeing with the ‘mind's eye’) and sensory perception (Cichy, Heinzle, & Haynes, 2012; Kosslyn, 1994; Pearson & Kosslyn, 2015) means that mental imagery has the capacity to simulate not only the perceptual details of hypothetical events, but also the experiential correlates of such experiences in an as‐if‐real manner (Lang, 1979; Moulton & Kosslyn, 2009). Future cognition is thus key to planning behaviour, with evidence showing that imagery representations of emotion stimuli can evoke emotional response at subjective, physiological, and neural levels (Ji, Heyes, MacLeod, & Holmes, 2016). Simulations that yield pleasure or reward may increase approach behaviour (Renner, Ji, Pictet, Holmes, & Blackwell, 2017), whereas negative simulations may encourage avoidance (Kosslyn, Ganis, & Thompson, 2001; Moulton & Kosslyn, 2009). Given that disruptions to future cognition thus have the potential to impact everyday behaviour and that ABI may be vulnerable to disruptions in future cognition due to damage to executive prefrontal and other key brain structures (Pearson, Naselaris, Holmes, & Kosslyn, 2015), this is an important area to investigate. Difficulty anticipating future events, for example, could result in reduced planning and engagement in experiences and activities, including those that could be unexpectedly positive, with the resulting poverty of rewarding experience serving to encourage avoidance and maintain low mood.

Future thinking has been studied using various tasks. The Future Fluency Task (FFT; MacLeod & Byrne, 1996; MacLeod *et al*., 1997) requires participants to generate examples of positive and negative events that are likely or plausible in their personal futures, with how readily positive versus negative examples come to mind used to infer relative frequency. In non‐ABI samples, depression has been linked to decreased positive relative to negative fluency (MacLeod & Byrne, 1996; MacLeod *et al*., 1997). The Prospective Imagery Test (PIT; Stöber, 2000) assesses participants’ ability to generate mental imagery for possible positive and negative future scenarios (e.g., someone will ‘admire’ or ‘reject’ you), rating each for vividness. Consistent with the future fluency findings, individuals with clinical (Morina *et al*., 2011) and self‐reported (Holmes, Lang, Moulds, & Steele, 2008) depression report reduced vividness for imagined positive, but not negative, scenarios, relative to controls.

Here, we investigate whether a similar association between future‐oriented cognition and depressed mood is evident following ABI, where more general deficits in cognitive function are common. The FFT and PIT were administered to individuals with ABI self‐reporting minimal to severe depressed mood. If a bias in future‐oriented thoughts and imagery is associated with depressed mood, this could provide an important basis for the development and application of timely and empirically derived interventions for depression following ABI. A sample of matched non‐ABI controls was also assessed to examine whether any ABI‐linked difficulties in generating and imagining future events remained apparent against background cognitive difficulties that might exacerbate, or indeed obscure, any biases in future cognition.

Our hypotheses were as follows:

1. On the FFT, ABI participants with self‐reported depressed mood would show a reduced positive bias relative to non‐depressed ABI and non‐ABI participants – due to reduced anticipated positive events, elevated anticipated negative events, or both.
2. A similar pattern would be evident on the PIT: depressed mood post‐ABI would be associated with a reduced positive bias relative to non‐depressed ABI and non‐ABI participants, reflecting a diminished ability to vividly imagine a positive versus negative future.

## Method

Ethical approval for this study was received from the Cambridge Psychology Research Ethics Committee. All participants provided prior written informed consent and were paid at the rate of £6 per hour. Demographic and clinical characteristics are reported in Table 1.

**Table 1:** Demographic details, mood assessment, and background cognitive performance data for ABI and control participants. ABI data are broken down by depression status (depressed versus non‐depressed), based on PHQ‐9 scores

Participants	All ABI (*n* = 40)	1. Dep ABI (*n* = 17)	2. Non‐dep ABI (*n* = 23)	3. Control (*n* = 30)	Pairwise *post hoc* test *p*‐values*		
					1 vs. 2	1 vs. 3	2 vs. 3
Sex (f:m)	20:20	13:4	7:16	15:15			
Age	59.1 (13.4)	55.4 (16.1)	61.8 (10.6)	60.0 (10.7)			
Years education	14.5 (3.0)	15.1 (3.1)	14.1 (3.0)	15.3 (3.1)			
NART‐IQ	112.2 (9.6)	113.5 (9.3)	111.3 (9.9)	114.7 (7.5)			
PHQ‐9	7.3 (6.4)	13.2 (4.7)	3.0 (3.4)	1.7 (2.0)	**<.001**	**<.001**	.38
BDI‐II	15.5 (11.1)	25.8 (8.4)	7.8 (5.0)	3.5 (3.8)	**<.001**	**<.001**	**<.05**
HADS Depression	6.0 (3.6)	8.6 (2.8)	4.1 (2.9)	2.1 (1.8)	**<.001**	**<.001**	**<.05**
HADS Anxiety	7.7 (4.2)	10.6 (4.2)	5.5 (2.6)	3.5 (2.7)	**<.001**	**<.001**	.08
PANAS Positive	3.1 (0.9)	2.7 (0.9)	3.4 (0.8)	3.7 (0.9)	**<.05**	**<.001**	.46
PANAS Negative	2.1 (0.8)	2.8 (0.7)	1.6 (0.4)	1.6 (0.5)	**<.001**	**<.001**	.99
Cattell correct	26.1 (6.2)	27.1 (6.9)	25.4 (5.7)	32.3 (5.0)	.73	**.01**	**<.001**
VFT (total correct)	37.7 (14.7)	41.9 (17.3)	34.8 (12.1)	49.6 (13.1)	.32	.22	**<.01**

Notes

Apart from sex, scores are mean (*SD*) values.

BDI‐II = Beck Depression Inventory 2nd Edition; Cattell = Cattell Culture Fair IQ Test; HADS = Hospital Anxiety and Depression Scale; PANAS = Positive and Negative Affect Schedule; NART = National Adult Reading Test‐Revised; PHQ‐9 = Patient Health Questionnaire‐9; VFT = Verbal Fluency Test.

*Sidak corrections have been applied to *p*‐values for all *post hoc* tests, with *p *<* *.05 in bold.

### Participants

#### ABI participants

Forty individuals (20 women) were recruited from the Cambridge Cognitive Neuroscience Research Panel (CCNRP) of individuals with ABI who have volunteered to take part in research. Depression status and/or mood symptom scores were available for a subset of members of the CCNRP. To ensure our sample included at least some individuals experiencing depressed mood, all individuals with existing mood information (including those with minimal depression symptoms) were invited to participate, accounting for approximately two‐thirds of our sample. To increase the sample size further, the remaining participants were recruited without any *a priori* knowledge of their mood status. The final group was split *post hoc* on the basis of Patient Health Questionnaire‐9 (PHQ‐9) depression scores, as described below. All participants reported ongoing difficulties due to their brain injury in one or more functional domains, for example cognition, movement, or affect. The sample ranged between 20 and 76 years, with a mean of 59.1 ± 13.4 years, and had an average post‐injury time of 10.1 ± 4.8 years. Brain injuries were due to cerebrovascular aetiology (stroke; *N* = 30), lesions resulting from resected tumours (*N* = 8), and tumour + stroke (*N* = 2). Twenty‐eight individuals had right‐sided, ten left‐sided, and two bilateral damage in frontal, temporal, parietal, and occipital cortex, as well as insula, thalamus, and basal ganglia.

#### Healthy control participants

Thirty age‐ and gender‐matched healthy controls (15 women) were recruited from the MRC Cognition and Brain Sciences Unit (CBU) non‐ABI volunteer panel. The sample ranged between 27 and 73 years of age (60.0 ± 10.7 years). Neither female:male ratio (χ^2^ = 0, *ns*) nor age (*t*(68)<1, *p *>* *.5) differed across the samples. ABI and control participants were additionally matched for *premorbid* cognitive ability (*F *<* *1, *p *=* *.36), indexed using the National Adult Reading Test (NART‐R; Nelson & Willison, 1991), which requires participants to read aloud 50 irregularly spelled English words that once acquired, are considered resistant to ABI.

### Mood assessment

The following depression and mood measures were administered, with scores reported in Table 1.

#### PHQ‐9 (Spitzer, Kroenke, & Williams, 1999)

The PHQ‐9 has 9 items that are based on the Diagnostic and Statistical Manual for Mental Disorders *(DSM‐IV‐TR*; APA, 2000) diagnostic criteria for major depression, with items scored 0 to 3. Defining depression in ABI using criteria developed in the non‐ABI population is complex because disturbances in energy, concentration, and sleep etc. may be attributable to neurological damage, depression, or both. However, the PHQ‐9 shows good test–retest reliability, internal consistency and construct validity, in ABI (Fann *et al*., 2005; Turner *et al*., 2012). Here, we followed recommendations for maximizing sensitivity and specificity in detecting depression in ABI (Fann *et al*., 2005) by employing a screening criterion of five or more symptoms (including at least one cardinal symptom – depressed mood or reduced interest/pleasure) for at least several days (i.e., score ≥1) in the most recent 2 week period. Seventeen ABI but no control participants were currently ‘depressed’ using this criterion; ABI group status based on this criterion provides the basis for all group comparisons (depressed ABI, non‐depressed ABI, and control).

#### Beck Depression Inventory‐II (BDI‐II; Beck, Steer, & Brown, 1996)

Twenty‐one self‐report items index depression symptoms over the past 2 weeks (e.g., sadness, concentration, and worthlessness), with the total ranging from 0 to 63. The BDI‐II has good concurrent validity and internal consistency in ABI (Turner *et al*., 2012).

#### Hospital Anxiety and Depression Scale (Zigmond & Snaith, 1983)

The Hospital Anxiety and Depression Scale (HADS; 7 depression/7 anxiety items, subscale scores between 0 and 21), which excludes somatic symptoms typical of other measures, has good concurrent validity and internal consistency in ABI (Turner *et al*., 2012).

#### Positive and Negative Affect Schedule – Short form (PANAS‐Short; Thompson, 2007)

The PANAS indexes negative *and* positive affect via its 5 negative and 5 positive items, each scored 1 *not at all* to 5 *extremely*.

#### Structured Clinical Interview for Depression (SCID; First, Spitzer, Gibbon, & Williams, 1996)

Although our focus was on self‐reported depressive symptomatology, to better characterize our sample we additionally determined current MDD diagnosis and history, according to the DSM‐IV‐TR (APA, 2000) using the Structured Clinical Interview for the DSM‐IV Axis 1 Disorders Clinician version (SCID; First *et al*., 1996). Eight ABI participants met diagnostic criteria for a current MD episode; 17 had a history of MD. No control participant had current MD, although seven reported a past episode.

### Background cognitive measures

#### Cattell Culture Fair Test, Scale 2, Form A (Cattell & Cattell, 1973)

The Cattell employs four novel timed non‐verbal problem‐solving tasks to index *current* intellectual function or ‘fluid’ intelligence. It is sensitive to deterioration of function due to age and neurological condition.

#### Verbal Fluency Task (VFT; Lezak, Howieson, & Loring, 2004)

Participants generated as many words as possible beginning with ‘F’, ‘A’, and ‘S’ in consecutive 1‐min trials, excluding proper nouns (people, places), words with the same root (e.g., swim, swimming), and numbers. Total *correct* items (excluding rule breaks) summed across the letters were computed. Widely used to measure mental flexibility and strategy generation, the VFT served here as a fluency control against which any specific difficulties in future cognition could be judged.

### Measures of future cognition

#### FFT (MacLeod *et al*., 1997)

Participants had 1 min to generate as many examples as possible of likely or plausible self‐future experiences, whether trivial or important, for each of three time periods – next week, next year, and next 5–10 years. These periods were included to facilitate performance over asking about ‘the future’ more generally (MacLeod *et al*., 1997). In a positive condition, participants generated examples of future experiences they would look forward to/enjoy. In a negative condition, they generated comparable examples of future experiences they were not looking forward to. Positive/negative order was determined at random. Total positive versus negative items were computed for each participant by summing across time periods.

Responses were transcribed by the experimenter and following the main task, read aloud to participants. Participants were asked to imagine and indicate how they would feel if events were to actually occur (1 *extremely unhappy* to 9 *extremely happy*), producing additional indices of anticipated emotion experience for positive and negative events.

#### PIT (Stöber, 2000)

Participants read and were asked to form a mental image for each of 10 negative and 10 positive future scenarios (e.g., ‘you will achieve the things you set out to do’), always presented in the same fixed order. Participants rated each mental image for (1) *vividness*, that is how clear or detailed it was (1 *no image* to 5 *very vivid*) and (2) *likelihood* in the near future (1 *not at all* to 5 *extremely* likely), following Morina *et al*. (2011).

### Procedure

Testing lasted approximately 3 hr. All but two ABI participants were tested in their homes; others were tested in dedicated MRC CBU testing space. Mood was assessed first, followed by background cognitive measures, and finally, prospective cognition measures. Individuals with ABI who were easily tired were assessed in two or more sessions. Additional measures (e.g., dysfunctional attitudes, rumination, autobiographical memory, and sustained attention) will be reported elsewhere.

## Results

### Statistical analysis

Data were analysed using SPSS Version 25 with alpha set at *p *=* *.05 and results of 2‐tailed tests reported. Group (depressed ABI, non‐depressed ABI, and control participants) comparisons were implemented using one‐way ANOVA. Emotion valence (positive, negative) was additionally incorporated as a within‐participant factor in 2‐way repeated‐measures ANOVA of FFT and PIT data. Sidak corrections were applied to *post hoc* tests (Field, 2013). Effect sizes were computed as eta‐squared (η^2^) or partial eta‐squared ($\eta_{p}^{2}$) for one‐way or repeated‐measures ANOVAs and Cohen's *d* for pairwise comparisons.

One ABI participant was unable to complete the VFT (language difficulties); another, the Cattell and PIT (comprehension difficulties). One control chose not to complete the FFT.

### Levels of mood disturbance and relationships between different measures

Significant group effects emerged for PHQ‐9 (*F*(2,67) = 71.92, *p *<* *.001, η^2^ *= *.68), BDI‐II (*F*(2,67) = 88.81, *p *<* *.001, η^2^ *= *.73), and HADS Depression (*F*(2,67) = 38.98, *p *<* *.001, η^2^ *= *.54), with higher scores in depressed than non‐depressed ABI participants and controls (*p*s <. 001; see Table 1). There were strong positive correlations in ABI participants between different depression symptom measures (PHQ‐9, BDI‐II, and HADS Depression; see Table 2). BDI‐II and HADS (but not PHQ‐9) depression scores were also significantly elevated in ‘non‐depressed’ ABI relative to control participants (*p*s < .05 for BDI‐II, HADS Depression; see Table 1). There was a significant group effect for HADS Anxiety (*F*(2,67) = 29.29, *p *<* *.001, η^2^ *= *.47) as well. Anxiety scores were higher in depressed than non‐depressed ABI participants and controls (*p*s < .001), and HADS Anxiety correlated with symptoms of depression (*p *<* *.001). Group effects also emerged for PANAS Positive (*F*(2,67) = 8.99, *p *<* *.001, η^2^ *= *.21) and PANAS Negative (*F*(2,67) = 34.51, *p *<* *.001, η^2^ *=*. 51) affect.

**Table 2:** Pearson correlations between measures of symptom severity and prospective cognition positive bias scores in ABI participants (*n* = 40)

	PHQ‐9	BDI‐II	HADS Depression	HADS Anxiety
BDI‐II	.85 (*p *=* *.000)*			
HADS Depression	.65 (*p *=* *.000)*	.70 (*p *=* *.000)*		
HADS Anxiety	.71 (*p *=* *.000)*	.81 (*p *=* *.000)*	.53 (*p *=* *.000)*	
FFT items	−.44 (*p *=* *.004)*	−.46 (*p *=* *.003)*	−.48 (*p *=* *.002)*	−.26 (*p *=* *.10)
PIT vividness	−.59 (*p *=* *.000)*	−.59 (*p *=* *.000)*	−.58 (*p *=* *.000)*	−.58 (*p *=* *.000)*

Notes

For FFT items generated, positive bias was calculated as the proportion of positive items out of all (positive + negative) items generated; thus, a higher proportion indicates a more positive bias (scores >.5 = positive bias, scores <.5 = negative bias). For PIT vividness, the bias score was calculated by subtracting ratings for negative scenarios from those for positive scenarios; a positive score thus indicates a positive bias, whereas a negative score indicates a negative bias. Given this, a negative correlation between bias scores for FFT or PIT indicates that as depression severity increases, positive bias on these tasks decreases, and *vice versa*. *p*‐Values in parentheses are uncorrected *p*‐values.

BDI‐II = Beck Depression Inventory 2nd Edition; FFT = Future Fluency Task; HADS = Hospital Anxiety and Depression Scale, PHQ‐9 = Patient Health Questionnaire‐9; PIT = Prospective Imagery Test.

*p‐Values that survive conservative Sidak correction for multiple comparisons.

### Background cognitive measures

#### Cattell

Current fluid intellectual function varied by group (*F*(2,66) = 10.5, *p *<* *.001, η^2^ *= *.24; see Table 1). This was due to both depressed and non‐depressed ABI participants having lower Cattell scores than controls (*p *=* *.01 and .001, *d = *.91 and 1.22, respectively), indicating a ‘loss’ of intellectual function, with no evidence for a difference between depressed and non‐depressed ABI participants (*p > *.5, *d = *.30). The pattern is consistent with the absence of significant correlations between PHQ‐9 and NART or Cattell scores across ABI participants (|*r*s| < .1, *p*s > .5).

#### VFT

A group effect emerged for verbal fluency (*F*(2,66) = 7.44, *p *=* *.001, η^2^ *= *.18; see Table 1). Consistent with ABI‐linked difficulties in executive function, non‐depressed ABI participants generated fewer correct examples than controls (*p *<* *.01; *d = *1.07)^1^; there was no evidence for a significant difference between depressed and non‐depressed ABI participants (*p *>* *.2, *d *=* *.51).

Thus, cognitive function is reduced in ABI relative to non‐ABI control participants, with no evidence for an association between background cognition and depression symptomatology within the ABI sample.

### Performance on tests of prospective cognition

#### FFT

**Figure 1:**
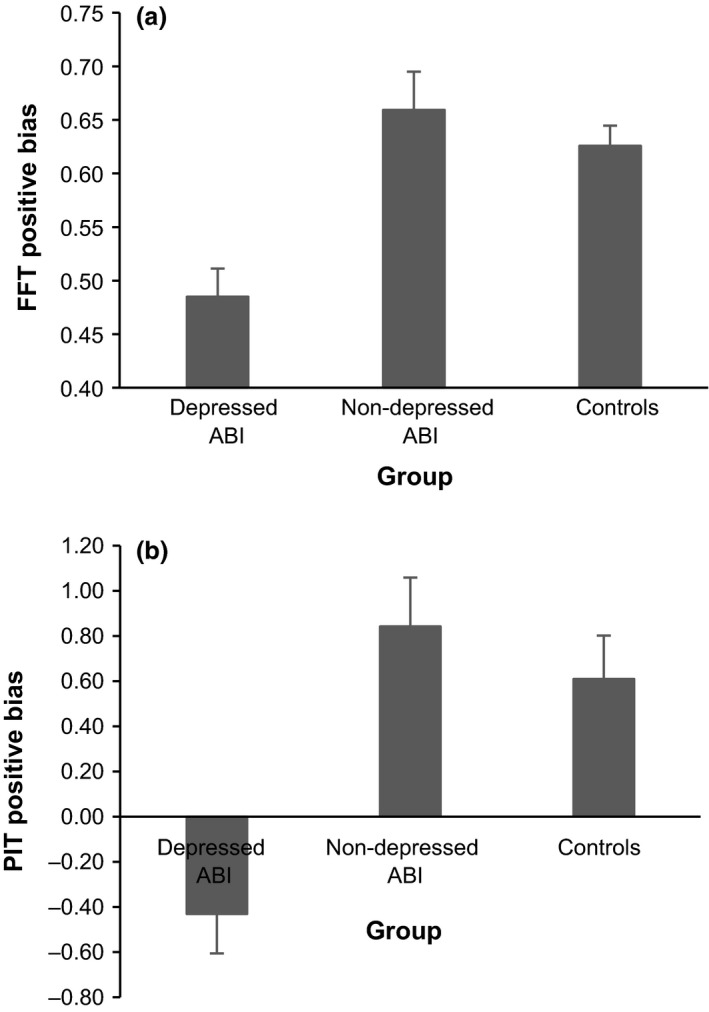
Positive bias scores in depressed ABI participants non‐depressed ABI participants and matched non‐ABI control participants for (a) items generated on the Future Fluency Task (FFT) and (b) vividness ratings on the Prospective Imagery Test (PIT). Data are mean values with error bars equivalent to 1 standard error of the mean. Positive bias was calculated on the FFT as a proportion (number of positive items divided by total positive and negative items generated) and on the PIT by subtracting vividness ratings for the negative from positive condition. Thus, higher/positive scores indicate positive bias. Data for positive and negative conditions separately are presented in Table 3.

Items generated and anticipated emotion are presented in Table 3. Analysis of generated items yielded a main valence effect, with participants overall generating more positive than negative examples (*F*(1,66) = 39.47, *p *<* *.001, $\eta_{p}^{2}$ = .37). The group effect was also significant (*F*(2,66) = 4.51, *p *<* *.05, $\eta_{p}^{2}$ = .12). Non‐depressed ABI participants generated fewer examples than controls (*p *=* *.017, *d *=* *.80). There was no evidence that depressed ABI participants and controls (*p *=* *.14, *d *=* *.61), or depressed and non‐depressed ABI participants (*p *>* *.5, *d = *.19), differed. These effects were qualified by a significant group × valence interaction (*F*(2,66) = 9.67, *p *<* *.001, $\eta_{p}^{2}$ = .23) which was due to greater positive versus negative fluency in controls (*p *<* *.001, *d *=* *1.34) and non‐depressed ABI participants (*p *=* *.001, *d *=* *.87) but not in depressed ABI participants (*p *>* *.5, *d *=* *.01), in whom there was no evidence for a discrepancy between positive and negative items (see Figure 1a).

**Table 3:** Performance data for ABI and control participants on the experimental measures of future cognition. Data for ABI participants are broken down according to depression status (depressed versus non‐depressed), as categorized by scores on the PHQ‐9

Participants	All ABI participants	Depressed ABI participants	Non‐depressed ABI participants	Control participants
FFT positive				
Number of items generated	14.2 (6.8)	12.9 (7.8)	15.1 (6.1)	20.8 (7.8)
Anticipated emotion	7.9 (.8)	7.8 (.8)	7.9 (0.7)	7.9 (0.5)
FFT negative				
Number of items generated	10.4 (6.9)	12.9 (6.2)	8.5 (6.8)	12.2 (5.5)
Anticipated emotion	2.0 (.9)	1.9 (.8)	2.1 (.9)	2.7 (0.7)
PIT positive				
Vividness	3.2 (0.9)	2.9 (0.9)	3.4 (0.8)	3.5 (0.8)
Likelihood	3.0 (0.8)	2.4 (0.7)	3.3 (0.7)	3.4 (0.7)
PIT negative				
Vividness	2.9 (0.8)	3.3 (0.8)	2.5 (0.5)	2.9 (0.9)
Likelihood	2.8 (0.9)	3.5 (0.7)	2.3 (0.6)	2.5 (0.7)

Notes

Data are mean (*SD*) scores.

FFT = Future Fluency Task; PIT = Prospective Imagery Test.

Given the group difference for VFT, future fluency was reanalysed, covarying for VFT (total correct items generated). The group × valence interaction remained significant (*F*(2,64) = 9.48, *p *<* *.01, $\eta_{p}^{2}$ = .23), although the group effect no longer achieved statistical significance (*F*(2,64) = 1.54, *p *=* *.22, $\eta_{p}^{2}$ = .05). The interaction, as described just above, was due to greater positive versus negative fluency in control and non‐depressed ABI participants but not in depressed ABI participants.

Anticipated emotion for generated future events was also analysed. Across participants, anticipated positive emotion was as expected, higher for positive than negative events (*F*(1,65) = 1421.1, *p *<* *.001, $\eta_{p}^{2}$ = .96). The group x valence interaction did not achieve significance (*F*(2,65)=2.35, *p *=* *.10, $\eta_{p}^{2}$ = .07), but there was a significant group effect (*F*(2,65) = 8.14, *p *=* *.001, $\eta_{p}^{2}$ = .2). Depressed and non‐depressed ABI participants produced less positive (i.e., more negative) estimations than control participants averaged across positive and negative events (*p = *.001 and .05, *d *=* *1.24 and .67, respectively) but with no evidence of a difference between depressed and non‐depressed ABI participants (*p *>* *.25, *d *=* *.53).

#### PIT

Vividness and likelihood ratings are presented in Table 3. Analysis of vividness ratings yielded a significant effect of valence, with participants on average reporting more vivid imagery for positive than negative scenarios (*F*(1,66) = 7.90, *p *<* *.01, $\eta_{p}^{2}$ = .11). This was qualified by a group x valence interaction (*F*(2,66) = 8.80, *p *<* *.001, $\eta_{p}^{2}$ = .21): vividness was higher for positive than negative scenarios in controls and non‐depressed ABI participants (both *p*s < .01, *d *=* *.58 and .61), but not in depressed ABI participants (*p *=* *.08, *d *=* *.61), where higher vividness ratings for negative scenarios approached significance (see Figure 1b). The group effect did not achieve significance (*F*(2,66) = 1.18, *p *>* *.3, $\eta_{p}^{2}$ = .04).

A group × valence interaction was similarly evident for likelihood ratings (*F*(2,66) = 19.7, *p *<* *.001, $\eta_{p}^{2}$ = .37). Whereas non‐depressed ABI and control participants (both *p*s < .01, *d *=* *.84 and .82, respectively) estimated a greater likelihood for positive versus negative future events, the reverse was true for depressed ABI participants, where estimates were higher for negative versus positive scenarios (*p *<* *.01, *d *=* *.94). Neither group (*F* < 1) nor valence (*F*(1,66) = 3.3, *p *=* *.08, $\eta_{p}^{2}$ = .05) effects achieved significance.

#### Power analyses

Given the modest sample sizes employed, statistical power was computed *post hoc*, using GPower3.1. For both the FFT and PIT, the current sample size (*N* = 69 overall) produced large effects on both tasks (see above), with 100% power to detect the interaction effects observed in our analyses, corresponding to *p *<* *.05. This significantly exceeds the minimum 80% power routinely sought in well‐powered studies, providing confidence in the reliability and generalizability of our findings.

### Relationship between symptom severity and prospective cognition bias scores in ABI participants

In the results above there is no evidence in the responses of depressed ABI participants for the positive bias that is present in non‐depressed ABI and control participants. Here, we additionally compute FFT and PIT bias scores for the purpose of conducting correlations with symptom severity scores across the ABI group as we wished to (1) rule out the possibility that the above findings were present simply for depressed and non‐depressed subgroups defined according to what are arguably *arbitrary* PHQ‐9 cut‐offs and (2) demonstrate the same pattern across *three* widely used measures of depression symptomatology (rather than just the PHQ‐9), thus enhancing confidence in the reliability and generalizability of our findings. To this end, positive bias was calculated (1) on the FFT as a proportion (number of positive items divided by total positive and negative items generated) and (2) on the PIT (vividness and likelihood) by subtracting ratings for the negative from positive condition. Thus, higher/positive scores indicate positive bias.

**Figure 2:**
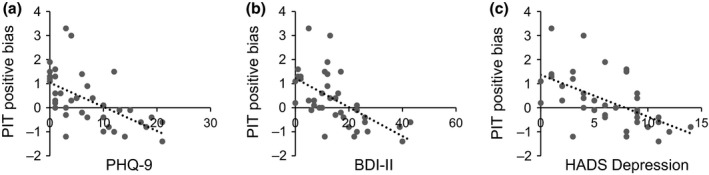
Correlations in ABI participants between positive bias scores for vividness ratings on the Prospective Imagery Test (PIT) and self‐report depression symptomatology indexed using the (a) PHQ‐9, (b) BDI‐II, and (c) HADS Depression subscale.

As seen in Table 2, positive bias scores correlated negatively with depression severity indexed via all three depression measures (PHQ‐9, BDI‐II, and HADS) and remained significant following conservative Sidak correction for multiple comparisons. The significant correlations between self‐report depression symptoms and positive bias on the PIT can be seen in Figure 2. HADS Anxiety correlated negatively with bias on the PIT but not FFT. Positive bias on the FFT and PIT also correlated significantly and positively for vividness (*r *=* *.62) and likelihood (*r *=* *.57). Thus, generating a greater proportion of positive future events was associated with more vivid imagery and increased anticipated likelihood for positive versus negative future events.

## Discussion

Mental health difficulties are common following ABI, with depression in particular having a negative impact on outcome, response to rehabilitation, and QoL. In the non‐ABI population, depression is characterized by relative negative biases in information processing. Here, we examined whether such biases are evident in the future cognition of individuals with ABI, where difficulties in attention, memory, and executive function can be present. To separate any affective bias from the more general effects of ABI, we compared ‘depressed’ and ‘non‐depressed’ individuals with ABI to healthy non‐ABI participants. The results were clear: whether generating likely future events or imagining future scenarios, the positive bias typically observed in healthy non‐ABI individuals, though evident in our non‐depressed ABI participants, was not evident in ABI individuals with elevated levels of self‐reported depression symptoms. These associations were corroborated in correlational analyses that demonstrated significant negative associations between the extent of positive bias on *both* future thinking tasks with self‐reports of depression severity indexed via each of three commonly used depression measures (PHQ‐9, BDI‐II, and HADS). These patterns emerged against a background of general cognitive‐executive impairment in the ABI group, relative to non‐ABI controls.

Participants were also asked to anticipate future experienced emotion for likely future events, ranging from extremely unhappy to extremely happy. All participants estimated more positive emotion for positive than for negative possible or likely future events, as would be expected. However, ABI participants estimated somewhat less positive (i.e., more negative) emotion than control participants for future events overall, with no evidence of a difference between depressed and non‐depressed individuals with ABI.

Future cognition can play an important role in the maintenance of low mood because of its likely effects on motivation and behaviour. Behavioural models posit that depression is associated with increased avoidance (e.g., social withdrawal) and decreased approach (e.g., engaging in fewer potentially rewarding behaviours such as physical activity) behaviour and that these play a central role in the development and maintenance of depression, and recovery (Lewinsohn, 1974). It is easy to see how reduced ability to generate examples of future events in ABI, and particularly potentially positive or rewarding ones in individuals experiencing depressed mood, alongside relatively lower imagined vividness and anticipated likelihood for positive versus negative future scenarios, could result in reduced levels of activity overall. It follows that individuals who are less likely to anticipate experiencing positive future events, or imagine them to be associated with reduced likelihood, will likely be less motivated to make them happen. Via such mechanisms, individuals may fail to experience reinforcing events that have the potential not only to lift their mood but also to challenge the generalized veracity of a negative worldview.

This cycle may be particularly difficult to shift in the context of ABI due to co‐occurring cognitive difficulties that are commonly present in ABI samples. These may include difficulties in making (Dritschel, Kogan, Burton, Burton, & Goddard, 1998) and remembering to follow through with plans at the appropriate time (Fish, Wilson, & Manly, 2010), poor attention (Hochstenbach, Mulder, van Limbeek, Donders, & Schoonderwaldt, 1998; Hyndman, Pickering, & Ashburn, 2007) to experience whilst engaging in positive events, reduced cognitive control (Larson, Perlstein, Demery, & Stigge‐Kaufman, 2006) that may make it difficult to resist depression‐related or ruminative thoughts and focus on the task at hand, impaired ability to abstract from the specific to the more general (Salas, Gross, Rafal, Viñas‐Guasch, & Turnbull, 2013), and poor episodic memory for completed activities, including positive aspects (Lim & Alexander, 2009). Added to this, ABI may be additionally associated with compromised executive function in the form of poor problem‐solving and fluid intelligence. Importantly, the depression‐linked disruptions in cognitive–affective processing observed here emerged against a background of more general difficulties in cognitive function in our entire ABI sample – in verbal fluency, future fluency, and executive function. Thus, in addition to depression‐linked disruptions in the protective positive bias present in the cognitive–affective processing of non‐depressed participants, ABI had a more general effect on cognitive function, compared to the general population, and despite being matched for premorbid intelligence. Importantly, these impairments were independent of extent of depression symptomatology in the ABI participants.

Whilst cognitive‐behavioural therapy (CBT) is an effective ‘talking therapy’ for depression in the general population (Cuijpers *et al*., 2013), its requirements may be particularly challenging for individuals post‐ABI. CBT can require people to recall incidents associated with low mood, keep negative automatic thoughts logs, consider antecedents and consequences, identify associated patterns of thinking, generate future strategies, and remember to implement these in everyday life. Each may be compromised by the post‐ABI language, memory and attention impairments, and difficulties in flexible cognitive‐behavioural adjustment (Broomfield *et al*., 2011) that are not inconsistent with the general cognitive impairments we observed here. A recent review of CBT for depression post‐ABI concluded that symptom reduction was typically partial and by no means apparent in all individuals (Waldron, Casserly, & O'Sullivan, 2013), and in a randomized controlled trial conducted at 3 and 6 months post‐stroke, there were no differences between CBT, an attention placebo, or routine care upon either depression severity or activities of daily living.

The current findings suggest that interventions that target future cognition may benefit individuals post‐ABI. In considering the cognitive difficulties that are common in ABI, Kneebone (2016) concluded that interventions emphasizing behavioural (rather than cognitive) techniques may produce better outcomes for depression following ABI. One such approach is behavioural activation (BA)(Martell, Dimidjian, & Herman‐Dunn, 2010), which seeks to schedule relevant and positively reinforcing activities. Meta‐analyses in non‐ABI individuals demonstrate BA is not only a highly effective low‐intensity intervention for depression (Cuijpers, van Straten, & Warmerdam, 2007; Cuijpers *et al*., 2011; Ekers *et al*., 2014) but also non‐inferior to CBT (Richards *et al*., 2016). It is thus encouraging to see studies beginning to apply BA following ABI (Lincoln & Flannaghan, 2003; Thomas, Walker, Macniven, Haworth, & Lincoln, 2012) and showing promise at least as applied to stroke, perhaps particularly in those with aphasia. Added to this, our findings indicate that BA may benefit from adaptation if it is to be optimally applied in individuals who have sustained a brain injury.

The current findings emphasize the importance of considering not just the altered depression‐linked biases in cognitive–affective processing but also more basic impairments associated with ABI in ‘cold’ executive and other cognitive functions that include but are not limited to future thinking. These more basic difficulties – that can sometimes be exacerbated in those with depression – may interact with cognitive–affective processing biases to maintain episodes of depressed mood and hinder opportunities for recovery in ABI. The effective delivery of BA might thus require additional structure and scaffolding in individuals post‐ABI, with additional support needed, for example, in the generation of examples of activities deemed appropriate for activity scheduling. This might be the case particularly in individuals for whom physical or language barriers pose additional obstacles to engagement, or where simulations of possible future activities are characterized by reduced vividness and likelihood – especially when this is for activities that might have potentially positive or mood‐lifting outcomes.

The current results indicate an association between depressive symptoms in ABI and a lack of positive *bias* in future‐oriented thinking, rather than simply an impairment *per se*. Given the paucity of existing research investigating depression‐linked cognitive biases in individuals with ABI, our study employed three different depression measures (PHQ‐9, BDI‐II, and HADS). Observing a similar pattern of association for each of these scales thus increases confidence in the reliability and generalizability of our findings which suggest that behavioural approaches and interventions currently being developed to target such biases in depression outside the ABI context (that is, in non‐ABI populations) that involve repeated practice in positive imagery generation may also have potential application in ABI populations. Consistent with the links between imagery, anticipated reward, and behaviour outlined above, there is preliminary evidence that computerized cognitive training procedures involving repeated generation of positive mental imagery could increase activation and reduce anhedonia in the context of depression (Blackwell *et al*., 2015; Renner *et al*., 2017). Such cognitive training approaches could conceivably supplement other interventions for depression in ABI such as BA. As for BA, however, they would likely require adaptation and their applicability would of course vary according to the specific nature of the individual's ABI.

To our knowledge, this is the first study of prospective biases in information processing associated with depression in individuals who have sustained a brain injury. These findings provide an important first step in understanding processes that may play a role in the onset and maintenance of depression in ABI. There are of course limitations to the generality of inferences that we can draw. The size of the ABI participant group was relatively small, although *post hoc* power analyses demonstrated our samples had excellent power to detect the interaction effects observed in our analyses. Self‐report depression symptom scales were administered before the future cognition tasks and so ruling out the possibility that completion of the depression symptom measures may have primed prospective cognition requires further study. There is also the issue of what we mean by ‘bias’. ‘Bias’ is used here in the rather limited sense of reflecting performance that differs across the positive vs. negative conditions of our prospective cognition tasks. It is possible that the absence of evidence for a positive bias in our depressed ABI participants for both imagining and generating examples of future events could be an accurate (rather than inaccurate) reflection of differing life circumstances in which depressed ABI participants may in fact have fewer positive relative to negative life experiences. Ascertaining whether the current depression‐linked biases in future cognition following ABI are accurate or inaccurate reflections of participants’ personal futures is important and necessitates careful future study. Our ABI participants were generally a long period post‐injury and sufficiently motivated to join a neuroscience volunteer panel. It will be important to investigate whether similar biases are evident in ABI participants with more recent injuries and more profound cognitive impairment. Whilst unpacking causal links is difficult, not least because symptoms attributed to ABI feature in depression and *vice versa*, it is of clinical importance to gain scientific traction on these issues due to the increasing availability of evidence‐based treatments that can offset low mood in the general population. Demonstrating similarities in the cognitive–affective processes relevant to depressed mood in ABI and in the non‐ABI population thus holds promise for improving outcome and furthermore suggests potential new routes for reducing depression following brain injury.

## Ethical standards

The authors assert that all procedures contributing to this work comply with the ethical standards of the relevant national and institutional committees on human experimentation and with the Helsinki Declaration of 1975, as revised in 2008.

## Footnote

1 For interest, previous reports (Roca *et al*., 2010) indicate that a significant proportion of verbal fluency performance variance can be accounted for by general intellectual function and VFT clearly also requires word knowledge. This was true of the ABI sample here, where VFT correlated significantly with both Cattell and NART performance (*p *< .05 in both cases).
